# RETRACTION: The long noncoding RNA AK002107 negatively modulates miR‐140‐5p and targets TGFBR1 to induce epithelial‐mesenchymal transition in hepatocellular carcinoma

**DOI:** 10.1002/1878-0261.13677

**Published:** 2024-06-14

## Abstract

RETRACTION: Tang, Y. H., He, G. L., Huang, S. Z., Zhong, K. B., Liao, H., Cai, L., Gao, Y., Peng, Z. W., & Fu, S. J. (2019), The long noncoding RNA AK002107 negatively modulates miR‐140‐5p and targets TGFBR1 to induce epithelial‐mesenchymal transition in hepatocellular carcinoma. *Mol Oncol*, 13(5): 1296–1310. https://doi.org/10.1002/1878-0261.12487.

The above article, published online on 11 April 2019 in Wiley Online Library (wileyonlinelibrary.com), has been retracted by agreement between the journal Editor‐in‐Chief, Kevin Ryan, FEBS Press, and John Wiley and Sons Ltd. The retraction has been agreed following an investigation into concerns raised by a third party, which revealed image duplication between figure 2D of this manuscript and figure 4G of an earlier publication by different authors [1]. The authors provided images of the trays of cell colonies which match the panels in figure 2, but did not provide a compelling explanation for the image duplication with the earlier publication [1]. Therefore, the editors consider the conclusions of the study to be substantially compromised and are retracting the article. The authors do not agree with the retraction.

Reference

[1] Zeng JD, Zhang N, Zhao GJ, Xu LX, Yang Y, Xu XY, Chen MK, Wang HY, Zheng SX, Li XX. (2018) MT1G is Silenced by DNA Methylation and Contributes to the Pathogenesis of Hepatocellular Carcinoma. *J Cancer*, 9(16): 2807–2816. https://doi.org/10.7150/jca.25680

RETRACTION: Tang, Y. H., He, G. L., Huang, S. Z., Zhong, K. B., Liao, H., Cai, L., Gao, Y., Peng, Z. W., & Fu, S. J. (2019), The long noncoding RNA AK002107 negatively modulates miR‐140‐5p and targets TGFBR1 to induce epithelial‐mesenchymal transition in hepatocellular carcinoma. *Mol Oncol*, 13(5): 1296–1310. https://doi.org/10.1002/1878-0261.12487.

The above article, published online on 11 April 2019 in Wiley Online Library (wileyonlinelibrary.com), has been retracted by agreement between the journal Editor‐in‐Chief, Kevin Ryan, FEBS Press, and John Wiley and Sons Ltd. The retraction has been agreed following an investigation into concerns raised by a third party, which revealed image duplication between figure 2D of this manuscript and figure 4G of an earlier publication by different authors [1]. The authors provided images of the trays of cell colonies which match the panels in figure 2, but did not provide a compelling explanation for the image duplication with the earlier publication [1]. Therefore, the editors consider the conclusions of the study to be substantially compromised and are retracting the article. The authors do not agree with the retraction.

**Reference**

[1] Zeng JD, Zhang N, Zhao GJ, Xu LX, Yang Y, Xu XY, Chen MK, Wang HY, Zheng SX, Li XX. (2018) MT1G is Silenced by DNA Methylation and Contributes to the Pathogenesis of Hepatocellular Carcinoma. *J Cancer*, 9(16): 2807–2816. https://doi.org/10.7150/jca.25680

